# The Role of Chosen Essential Elements (Zn, Cu, Se, Fe, Mn) in Food for Special Medical Purposes (FSMPs) Dedicated to Oncology Patients—Critical Review: State-of-the-Art

**DOI:** 10.3390/nu15041012

**Published:** 2023-02-17

**Authors:** Adrian Frydrych, Mirosław Krośniak, Kamil Jurowski

**Affiliations:** 1 Laboratory of Innovative Toxicological Research and Analyses, Institute of Medical Studies, Medical College, Rzeszów University, Aleja Majora W. Kopisto 2a, 35-959 Rzeszow, Poland; 2 Department of Food Chemistry and Nutrition, Medical College, Jagiellonian University, Medyczna 9, 30-688 Kraków, Poland; 3 Department of Regulatory and Forensic Toxicology, Institute of Medical Expertises, Aleksandrowska 67/93, 91-205 Łódź, Poland

**Keywords:** food for special medical purposes, oncology, cancer, essential elements

## Abstract

The scoping review aimed to characterise the role of selected essential elements (Zn, Cu, Se, Fe, Mn) in food for special medical purposes (FSMPs) aimed at oncology patients. The scope review was conducted using Scopus, Google Scholar, and Web of Science to find published references on this subject. Data from the reviewed literature were related to the physiological functions of the element in the body, and the effects of deficiencies and excesses, referring to the latest ESPEN and EFSA guidelines, among others. Important dietary indices/parameters based on the literature review are provided for each element. On the basis of the literature, data on the level of elements in patients with cancer were collected. The content of these elements in 100 mL of FSMPs was read from the manufacturers’ declarations. The literature has been provided on the importance of each element in cancer. Our findings show that the essential elements (Zn, Cu, Se, Fe, and Mn) of FSMPs for cancer patients are not adequately treated. We suggest solutions to ensure the safe use of FSMPs in oncology patients.

## 1. Introduction

Nutrition science deals with all aspects of the interaction between food and nutrients, life, health, and disease, and the processes by which an organism ingests, absorbs, transports, uses, and excretes food substances [1,2]. The field of medical nutrition, or, actually, clinical nutrition, is evolving rapidly, and several problem areas await solutions through future research. The provision of nutritional support to compromised patients is a challenge that can only be met through joint efforts between the scientific and medical community and innovative industrial leaders (pharmaceutical industry). From this point of view, in particular, imports are products available in pharmacies/drug stores (i.e., food for special medical purposes (FSMPs)) [3]. Based on the directive, it repeals Directive 1999/21/EC [4] (a delegated act that supplements Regulation (EU) No. 609/2013 on foods for specific groups), so FSMPs:

- May only be placed on the market if it complies with this regulation;
- Falls within three categories:
  ○ Nutritionally complete food with a standard nutrient formulation, which could constitute the sole source of nutrition, or be used as a partial replacement or supplement;
  ○ Nutritionally complete food with a formulation adapted to nutrient requirements specific for a disease, disorder, or medical condition, which could constitute the sole source of nourishment or be used as a partial replacement or supplement;
  ○ Nutritionally incomplete food that is not suitable for use as the sole source of nutrition.

Several FSMPs have been reported to have significant pain relief efficacy with multiple antioxidant and anti-inflammatory properties. FSMPs are especially important for children; milk formulas for healthy children who cannot be breastfed can be divided into two groups: the initial, that is, the initial (first) milk formula from birth to 6 months, and the subsequent (follow-up) milk formula used from the age of 7 months to 1 year [5,6]. However, it is also important for adults, especially oncology patients [7]. It should be underlined that FSMPs for patients are generally regulated as a pharmaceutical product. Therefore, they should be available in pharmacies/drug stores due to the special conditions of quality, safety, production, and distribution [8,9].

The traditional diet of oncological patients often does not provide a sufficient supply of elements. To improve the supply of elements and kilocalories, the therapeutic team introduces food for special medical purposes (FSMPs) that are available in pharmacies in the diet of cancer patients. FMSP manufacturers declare the content of elements in their products (e.g., zinc, cooper, selenium, iron, and manganese) [10]. However, there are no experimental studies that have confirmed the participation and adequate bioavailability of elements in FSMPs. Furthermore, these elements are not properly treated by the manufacturer (the levels are not correlated with the existing scientific data on cancer). The excess and deficiency of elements in the supply of FSMPs should be considered in the case of exclusive PN nutrition, as should the nutritional status of the patients and the possible side effects that occur when the homeostasis of elements in the body is disturbed. Another important element of the possibility of a faster cure is the behaviour of body homeostasis in inflammation associated with cancer. Data linking the specified elements with cancer-free FSMPs are scarce and there is a lack of adequate scientific literature on the role of essential elements in FSMP dedicated to cancer patients, that is, cancer nutrition that includes essential elements such as copper, manganese, zinc, iron, and selenium. Therefore, this critical review aims to provide a coherent overview of the literature (scientific literature and grey literature) on the role of essential elements (Cu, Fe, Mn, Se, and Zn) in FSMPs dedicated to oncology patients.

## 2. Materials and Methods

### 2.1. Search for Publications on the Content and Role of Essential Elements in FSMPs Dedicated to Cancer Patients

The three main repositories for locating published references on this topic are Scopus, Google Scholar, and Web of Science, which were used for a critical examination of the key components in the context of FSMPs for cancer patients. It should be emphasised that in addition to the previously specified scientific sources, the ‘grey’ literature was also searched during the data collection procedure (especially the declarations of the manufacturers). 

### 2.2. Keywords and Selection of Scientific Data

Different combinations of the following main terms were used: FSMP, oncology nutrition, elements of the oncology diet, oncology diet, clinical nutrition in oncology, food for medical purposes for cancer, and a combination of the mentioned terms. Two steps were applied to the selection of the studies: (1) selection by title and abstract and (2) full text examination. The selection of the title and abstract was performed independently by each author at different times. Included and excluded studies were critically identified after defining the problem formulation. Studies that met the eligibility criteria were kept for the next screening step. Studies clearly not relevant to the problem formulation or exclusion criteria were excluded. We analysed all available sources (n = 216 articles and related content). To filter the sources retrieved, only articles/works related to the content or role of essential elements (Cu, Mn, Zn, Fe, and Se) in FSMPs dedicated to oncology patients. The authors then performed a full text deep examination of all of the important manuscripts.

### 2.3. Classification and Presentation of the Results

All information from the literature including relevant parameters (element concentrations in the serum of oncological patients, important dietary indices/parameters for Cu, Mn, Zn, Fe, and Se, and the content of elements in FSMPs) are reported in the present review. The role of each element in the diet during cancer was extracted and systematically reported in the manuscript. 

### 2.4. Presentation of Results

For appropriate readability, we present the information in three paragraphs for each element: (1) Short part of the characteristics of the physiologically/biologically important properties of each investigated element; (2) The role as a potential biomarker of each type of cancer with levels in the biological samples; and (3) The role and level in the FSMPs available in pharmacies. When other relevant information (e.g., ESPEN recommendations) was known, it was presented in the last paragraph.

## 3. Results

### 3.1. Zinc

Zn is essential for the growth and development of microorganisms, animals, and plants. In the human body, zinc meets three general functional classes: regulatory, catalytic, and structural [11]. Zinc is essential as a catalytic, structural, and regulatory ion and is involved in the regulation of homeostasis, oxidative stress, immune responses, apoptosis, and ageing [12]. It contributes directly or indirectly to the transformation of proteins, fats, carbohydrates, and energy transformation. Zinc is necessary for the production and/or operation of multiple hormones. Zn also has an anticancer function due to its role in the structural stabilisation of DNA, RNA, and many transcription factors and proteins [13,14]. Zinc is responsible for ensuring the stability of cell membranes, alcohol metabolism, taste and odour, and immune defences of the organism [15,16]. Indirectly, it can participate in learning and learning processes and regulate signal stimulation and signal conduction in the central nervous system [16,17]. This metal contributes to the regulation of chronic inflammation by reducing pro-inflammatory cytokines. Zinc reduces oxidative stress by participating in the synthesis of antioxidant enzymes and acts as a catalyst for enzymes in the metabolism of carbohydrates, lipids, and proteins [15,18]. This element is involved in insulin storage, synthesis, and release, suggesting the key role of this microcomponent in the progression of type 2 diabetes, atherosclerosis, and metabolic syndrome (MS) [19]. The specific effect of zinc deficiency is observed in infants with enteropathy skin inflammation. The slowdown in growth is one of the best-defined effects of chronic zinc deficiency. Furthermore, zinc is essential for the immune system, and the lack of zinc affects many aspects of the immunity system and adaptability. The immunological changes during ageing and zinc deficiency exhibit remarkable parallels including a reduction in the activity of the thyroid and thyroid hormones, a shift from T-helper cells to type 2 T-helper cells, a decrease in vaccination response, and an impairment in the functions of the primitive immune cells [20]. Most zinc (85%) in the human body is found in the skeletal muscle and bones. A very small amount (about 0.1–1% of total) is in plasma, where 70% Zn is associated with albumin, and the concentration is correlated at approximately 10–17 mmol/L [11,21]. The reference value for Zn is approximately 60–120 µg/dL. Rats exposed to carcinogens (e.g., benzene, nitrosamines) were found to have a Zn deficiency in their diet, leading to increased susceptibility to cancer development [22,23]. The effectiveness of Zn supplements in the prevention of prostate cancer is controversial. Zinc supplements can help in the early stages of cancer development rather than during treatment [24]. During zinc treatment, stabilisation in the percystic adenomatosis levels of the wild colorectal bowel was observed at the post-transcriptional level [25], and a decreased contribution to the decrease in lymphocytes and erythrocytes with clinical symptoms as well as considered important deficiencies [26]. The value of zinc in serum is correlated with the serum zinc gastrointestinal tumour, and it was found that the serum zinc levels were more closely related to advanced gastric tumours [27]. Changes in zinc concentration were detected in patients with breast cancer. The Cu/Zn ratio can be used as a biochemical marker in such patients [28]. Acute acquired zinc deficiency conditions have also been documented, mainly in patients dependent on zinc-free intravenous nutrition [29]. Optimal levels of zinc in the body can also help reduce risk factors for cancer development, but this requires further study [30]. Zinc homeostasis disorders have been associated with oesophageal tumours, gastric cancers, colon cancer, and hepatocellular carcinoma [30]. It should be noted that the reference in Pasha et al. showed higher zinc values in patients with gastrointestinal tumours [31]. Table 1 shows the levels of zinc in the blood serum of patients with various cancers.

Organic forms of Zn (e.g., from oysters) are better absorbed when inorganic forms are added to the diet as a supplement [40,41]. Currently, enough is known about the clinical and public importance of zinc deficiency to establish beyond any reasonable doubt the exceptional practical importance of this trace element in human nutrition [42]. The average dietary zinc demand (AR) necessary to meet the physiological requirements was estimated by modelling the saturation response, considering the inhibitory effect of the dietary phytic on the absorption of Zn. Among the factors that predispose a person to zinc deficiency are cereal diets rich in phytates, malnutrition, alcoholism, anorexia nervosa, and ageing [16]. The estimated AR and the population reference intake (PRI) are given for levels of phytinate intake of 300, 600, 900, and 1200 mg/day. They cover the range of average consumption observed in the populations of European countries. The AR ranges from 6.2 to 10.2 mg/day for women of 58.5 kg and from 7.5 to 12.7 mg/day for men of 68.1 kg. PRI has been derived from the requirement for zinc for men of 97.5 percentile body weight for men and women of reference masses in the range 7.5 to 12.7 mg/day for women and 9.4 to 16.3 mg/day for men [43]. Available studies show that the mean zinc intake for adults and children in EU countries is below the acceptable upper intake level (UL). The 97.5 percentile of the total zinc intake for all age groups is close to the cut-off point, which the Committee believes is not of concern [43]. Table 2 shows the values/indices proposed/estimated for zinc.

The zinc provided can help protect against the inflammation of the oral mucosa and pharynx caused by radiotherapy [49]. Subamanyam et al. observed that patients treated with chemotherapy had elevated levels of zinc in serum [50]. In the study by Anandhi et al. [49], there was a trend toward less loss of taste and dryness in the mouth during radiation therapy, as well as oral pain associated with mucosal inflammation. No improvement was found in cases of mucosal inflammation caused by chemotherapy. Zinc does not affect the survival of cancer patients; among others, it will not reduce the effect of basic cancer therapy [51]. In the latest studies, patients with basal cell carcinoma were reported to have a lower zinc concentration than the control group [52]. Woźniak et al. showed that the highest concentration of zinc was observed in the hair in larynx cancer [53]. The Zn values for the study group–patients with cell carcinoma–were on average 78.65 μg/dL and in a healthy control group—89.39 ± 12.47 [52]. Other data in the literature indicated that zinc levels in gastrointestinal tumours have been decreased [54], serum or plasma zinc decreases have been observed in head and neck cancer [55], cervical cancer [56], lung cancer patients [57] and gynaecological cancers [58]. Serum zinc levels were correlated with gastrointestinal tumours and were found to be more closely related to advanced gastric tumours [27]. Additionally, the study demonstrated that zinc deficiency in cancer patients is correlated with disease development and negatively correlates with survival rates. Zinc showed exceptional cytotoxicity in both in vitro and in vivo, despite the small size of the samples. In the same study, zinc supplements were found to complement the zinc deficiency found in cancer patients and cancer drugs by managing cancer body homeostasis. Zinc is recommended to be part of cancer treatment [59]. Supplements and optimal zinc intake restore the correct immune response to goodbye of the immune system and reduce the risk of infection. At the same time, excess zinc has been shown to be dangerous due to its immunosuppressive effect, depletion of the resistance of the organism [60]. According to ESPEN Microelement recommendations (Recommendation 13.7) in acquired zinc deficiency, 0.51 mg/kg per day of elemental zinc (Zn^2+^), can be administered orally for 3–4 months [11]. According to our calculations, FSMP products contain approximately 1.2–1.8 mg of Zn/100 mL of products [10]. A summary of the Zn content declared in FSMP for oncological patients available on the EU markets is provided in Table 3.

The zinc content declared by producers in the products for special medical use for oncology patients is different. Individual FSMP contain 1.2 mg/100 mL Zn to 2.0 mg/100mL Zn. The most commonly used copper compound in FSMP is zinc sulphate [77,78].

### 3.2. Copper

Cu is another important trace element associated with cancer. In many studies, the deregulation of his homeostasis could be the cause and consequence of cancerogenesis due to its role in proliferation and angiogenesis [79]. The cellular biochemistry of copper in eucaryotes is diverse, as this element serves as an essential cofactor for many redox enzymes that react with oxygen and its reduced derivatives, for example, peroxide (O2^°2-^) [80]. These enzymes are involved in critical processes such as respiration (e.g., cytochrome c oxidase (C_c_O) [81], transfer of electrons/oxidation of the substrate and iron retention (ceruloplasmin), synthesis and metabolism of neurotransmitters (β-hydroxylamine, monooxygenase amidptydylogicin), pigmentation (tyrosinase) [82]. Copper is an essential element for all organisms that have an oxidative metabolism as a result of the rapid combination of the degree of oxidation. In the human body, copper is the third most abundant transitional metal [83]. Copper plays an essential role in various key enzyme systems and is closely related to normal haematopoiesis and cell metabolism. Furthermore, copper is associated with angiogenesis, hypoxia reaction, neuromodulation, and other biological processes [84]. In living matter, Cu has two levels of oxidation: Cu^+^ and Cu^2+^. Cu^2+^ is soluble, but the solubility of Cu^1+^ is in the submicromolar range. In biological systems, the bellows are in the form of Cu^2+^ because in the presence of oxygen or other electron acceptances, Cu^+^ easily oxidizes to Cu^2+^ [85]. Copper is considered an important micronutrient for all organisms. This crucial element plays an important role in mitochondrial respiration, photosynthesis, the electron transport chain, cell wall metabolism and lignin synthesis, and the response to oxidative stress and hormonal signalling in plants. Other functions performed by Cu in plants include carbon dioxide assimilation and ATP production [86]. The excess of intracellular copper may become cytotoxic. Genetic disorders such as Menks and Wilson disease (WD) illustrate the destructive importance of copper homeostasis in humans, which successively causes systemic copper deficiency or overload [87]. Symptoms caused by a copper deficiency in Menkes disease, e.g., affect the nervous system, development disorders, hypotonia, and severe intellectual disability [88]. WD is an autosomal recessive disease characterised by a deep cumulation of copper in the liver and several other organs (e.g., brain, kidney) due to toxicity (e.g., liver cirrhosis) [88]. Evidence suggests that brain imbalance of Cu can play a role in the pathogenesis of neurodegenerative disorders such as Parkinson’s disease, Alzheimer’s disease, and cardiovascular disease, sclerosis [89].

The proportion of copper in cancer has been studied for several decades and reports of abnormal levels of this element in neoplastic tissues have been reported in cancer patients [56]. Elevated levels of copper in serum were correlated with the stage of the disease and its progression in colorectal and breast cancer [90]. High levels of copper in serum were associated with various types of cancer, such as lymphoma, mesh cell sarcoma, bronchial and cervical cancer, breast, squamous larynx, stomach, and lung cancer [91]. The misalignment of Cu homeostasis in cancer patients has been well documented. Chronic exposure to elevated levels of Cu in drinking water has been shown to stimulate cancer proliferation in the neuroendocrine model of the pancreas [92]. High exposure to Cu increased the risk of prostate cancer [93]. Given its contribution to cancer proliferation, angiogenesis, and metastases, Cu is becoming a new target for cancer therapies. Recently, there has been significant progress in the perception of Cu in oncology therapy [94]. These observations have led to the hypothesis that the level of copper in the serum may be a biomarker of cancer relapse and can be measured to monitor the effectiveness of treatment [95]. Given the very limited evidence of the relationship between copper intake and cancer incidence, the data cannot be used to determine the value of DRV (dietary reference values) value for copper [96]. The clinical study reported that depleted Cu levels decreased markers of endothelial progenitor cells that are involved in metastases [97]. Copper levels should be measured in patients with long-term PN (parenteral nutrition), regularly every 6 to 12 months. In a clinical trial by Araya et al. which was observed in patients (n = 1600) with lung cancer, it was concluded that dietary intake of copper and zinc was associated with a reduced risk of lung cancer [98]. Copper-based drugs and signal paths are developed and tested. Parallelly, it is necessary to evaluate the functional ‘copper condition’ of patients to minimise side effects and any impact on basic biological processes [99]. The free serum copper is 10–15 μg/dL [100]. A summary of the critical review on Cu levels in different types of cancers as potential biomarkers is presented in Table 4.

The ESPEN recommendations state that enteric nutrition should supply 1–3 mg of copper per day at 1500 kcal. [11]. Copper ions (Cu^2+^) showed high reactivity with other elements of food (e.g., polyphenols) [107]. According to ESPEN, copper consumption in eight EU countries averages 1.47 to 1.67 mg per day (central value 1.57 mg/day) for healthy men and 1.20 to 2.07 mg/day for healthy women aged 18–< 65 years [96]. In the case of men, the panel proposes AI of copper at the level of 1.6 mg/day. For women, the panel proposes an AI of 1.3 mg/day [96]. The EFSA panel states that AR and PRI for copper cannot be derived for adults, infants, and children and proposes AI [96]. The relevant dietary indices/parameters for Cu are summarized in Table 5.

Increasing observations have combined copper signalling with cell proliferation as well as tumour growth and cancer metastases [99]. The influence of copper complexes on the prevention and treatment of cancer was studied by [110]. TM (tetrathiomolybdate), a highly specific copper chelator that was originally developed for the treatment of WD [111,112], has been used in studies in various combinations, for example, with chemotherapy in the clinical trial NCT01837329 [113] and 180 mg/day TM (40 mg with meals three times and 60 mg at bedtime) chemotherapy IFL (a chemotherapy regimen for certain cancers, consisting of simultaneous treatment of irinotecan), leucorine (folic acid), and fluorouracil [114]) [115]. Chelation-based treatments bind to copper and deactivate it [116]. Tumour growth and metastasis have a higher copper requirement [99]. Several field studies have shown a relationship between the increased prevalence of colorectal cancer (CRC) and an increased Cu level in serum compared to a healthy control group [117]. Serum levels of Cu, CP, ATP7A/7B, and the Cu:Zn ratio in serum are result-related markers and are resistant to treatment across the entire spectrum of gastrointestinal tumours including gastric and oesophageal cancers [98,99,100,101]. Expression of the Cu transporter is often increased in gastric cancer and can be used as a marker of resistance to chemotherapy against cisplatin in some patients with poorly differentiated or undifferentiated gastric cancer [118]. Copper depletion, as a therapeutic strategy, can direct many key processes essential in cancer evolution and the spread of metastases [119]. 

FSMPs contain approximately 0.18–0.25 µg Cu/100 mL of the product, depending on the composition and intended use [10]. A summary of the Cu content declared in FSMPs for oncological patients available on EU markets is shown in Table 6. According to the recommendations of the ESPEN micronutrient guidelines (Recommendation 6.3), feeding should be provided with 1–3 mg of copper per day at 1500 kcal [11].

As can be seen, the copper content declared by the manufacturers of FSMPs for oncology patients was not the same. According to the manufacturer’s data, these products contain approximately 0.13–0.09 mg Cu/100 mL of product [74,77,78,120,121,122,124,125].

### 3.3. Selenium

Selenium has chemical properties similar to those of sulphur, and to a lesser extent, tellurium. It occurs in four natural oxidation states: (0), elemental selenium, selenodiglutation (dipeptide); (-II), sodium selenite (Na_2_Se), hydrogen selenide (H2Se); (IV), sodium selenite (Na_2_SeO_3_), selenium dioxide (SeO_2_), selenium acid (H_2_SeO_3_); I (VI), sodium selenate (Na_2_SeO_4_), selenium acid (H_2_SeO_4_) [127]. Selenium is a trace element in many species including humans. This important element is incorporated into proteins through a specific selenium tRNA. Selenium is a component of oxidative enzymes and cytochrome. It participates in cell metabolism processes as part of glutathione peroxidase, an enzyme that protects cell membranes from damage by radicals and regulates the speed of peroxidase processes in cells. It contributes to the recovery of ascorbic acid from its oxidised metabolites (thioredoxin reductase) and is an essential ingredient for the metabolism of thyroid hormones [128,129]. Selenium, as a component of the new oxidative enzymes and cytochrome, is involved in cell metabolic processes [128]. It is part of the new glutatima peroxidase, an enzyme that regulates the speed of peroxidase processes in cells and protects cell membranes from damage by free radicals. As a component of another enzyme (tioredoxine reductase), it participates in the production of ascorbic acid from its oxidised metabolites. It is also necessary for the metabolism of thyroid hormones [130]. The reduced level of this component is found in AIDS and blood vessel diseases, phenylketonuria, acute pancreatitis, cystic fibrosis, retinopathy, renal failure, rheumatoid arthritis, and immune diseases and patients with depression [128].

This element protects against radicals by inhibiting tumour cell division and reducing the ability of carcinogenic compounds to induce cell mutations [131]. It has been studied for some cancers, but there is little scientific evidence of the relationship between Se in the diet (main source) and cancer risk [79]. By creating nonactive and nontoxic complexes, the use of selenium shows prophylactic effects in heavy metal intoxication (mercury, lead, cadmium, arsenic) [132]. Human exposure to selenium in various chemical forms can be achieved through food, drinking water, and air. Diet is the main source of human exposure to selenium [131]. Food primarily contains organic selenium, while an inorganic form is present in drinking water and air [133]. An example of selenium deficiencies is youth cardiomyopathy (Keshan disease) and cartilage dystrophia (Kashin-back disease) in China. In areas with selenium deficiency, an increase in mortality was observed due to cancer and cardiovascular disease. The protective role of this crucial element in lung cancer was demonstrated by a meta-analysis, which showed a decrease in cancer incidence with its consumption [134]. The level of selenium in the diet has been shown to be related to the expression of selenoproteins and affects the immune response through its effect on the secretion of interferon-γ and IL-6 [135]. It has been shown that TNRD1 selenoprotein is overexpressed with 1.5 times the change in lung cancer compared to adjacent normal tissue [136]. Selenium was experimentally tested for its potential impact on cancer therapy for prostate, breast, lung, oropharyngeal, colorectal, bladder, skin, leukaemias, uterine, and ovarian cancers. The authors of the review showed that Se is still highly questionable, is specific to tumours, and is dose-specific (antioxidant action) [137]. Se supplementation has been shown to increase the incidence of oesophageal cancer and its effect on tumour volume [138]. Other studies have shown that Se supplementation reduces stomach and lung cancer in patients with low serum selenium levels [139]. The reference range for plasma selenium is approximately 12 to 16 ng/mL [140]. Table 7 shows the selenium concentrations tested in patients with oncological diseases (serum levels).

This element may reduce the risk of some cancer patients through P450, enzymes in the liver that can be caused by selenium, leading to the detoxification of certain carcinogenic particles and a reduction in tumour growth [145]. Supplementation with 200 or 400 µg/day of selenium as selenised yeast reduced the risk of prostate cancer among men at high risk for the disease, according to a prostate specific antigen (PSA) level exceeding 4 ng/L, suspicious digital rectal examination, and a PSA velocity greater than 0.75 ng/mL/y [146]. There is no conclusive evidence to suggest that increasing selenium intake through diet or supplementation prevents cancer in humans [131]. Enteral nutrition should provide 50–150 mg of selenium per day with 1500 kcal [11]. According to ESPEN recommendations considering good enteral absorption and in the absence of contraindication, the enteral route can be used with doses starting at 100 mg/day [11]. In the analysis of the dose–response relationship in the selected study subgroup in the Hurst et al. (2012) study, there was a decreased risk of prostate cancer at plasma/serum selenium concentrations between approximately 135 and 170 μg/L. The EFSA Panel noted that the Cochrane review by Vencetti et al. concluded that the exposure to selenium was inversely related to the risk of tractor cancer of the bladder and prostate, and that there were no serum/plasma selenium concentrations associated with a reduced cancer risk [131]. The panel noted that there is no evidence from the intervention studies that doses of selenium of 200 μg per day taken in addition to selenium in the diet can prevent cancer in humans [128]. Adequate intake was established at 70 μg/day for adults [128]. Important dietary indices/parameters for Se are reported in Table 8.

Enteral nutrition should provide 50–150 mg of selenium per day with 1500 kcal [11]. According to Recommendation 12.7, given good intestinal absorption and in the absence of contraindications, the intestinal tract may be used in doses starting at 100 mg/day. For plasma selenium <0.4 mmol/L (30 mg/L), the intravenous route may be used for rapid correction: administration of up to 400 mg/day may be necessary for at least 7–10 days, and then the condition is rechecked [11]. A summary of the Se content declared in the FSMPs for oncological patients available on EU markets is presented in Table 9.

### 3.4. Iron

The biological versatility of iron is directly due to its capacity to undergo oxidation–reduction (redox) reactions. As a transition metal, iron possesses unpaired electrons that make it a versatile participant in redox reactions. Iron can exhibit a wide range of oxidation states, from II to VI, although iron is limited primarily to the ferrous (II), ferric (III), and ferryl (IV) states in biological systems [151]. In bone, the bone marrow is used to produce red blood cells. In addition, it participates in DNA synthesis and plays an important role in the control of bacteria and viruses by the immune system. It also affects cholesterol metabolism and promotes the detoxification of harmful substances in the liver [15,129,152]. If the gut is handled correctly, there is a risk of systemic overcharging with the nutrient iron. Chronic overcharging with iron may occur as a result of typical clinical conditions and genetic mutations, but there is no evidence that patients with hemochromatosis are exposed to an increased risk of iron overload. If the gut is handled correctly, there is a risk of systemic overcharging with the nutrient iron. Chronic overcharging with iron may occur as a result of typical clinical conditions and genetic mutations [152]. Iron absorption from an average diet is between 10% and 15% and increases two to three times in the case of Fe deficiency in the body. Other components of the diet—phytates, polyphenols, vegetable protein, and certain mineral substances (e.g., calcium, zinc)—can adversely affect the absorption efficiency of nonphemic iron. The presence of foods with a high vitamin C content in food has a positive impact on absorption [153]. 

The physiological mechanisms of iron handling change in cancer, which has been called an iron stop reaction, an attempt to limit the availability of this element to the tumour. This reaction is similar to that caused by bacterial infection [154]. Misalignment of iron homeostasis is often observed in patients with cancer and is indicated by a reduction in the number of red blood cells or anaemia [155]. Cancer cells often change iron metabolism in a way that promotes iron storage: towards the increased absorption and storage of iron, reduced exports of iron, or both. Increased iron accumulation is specific for tumour initiation cells [156] and neoplastic stem cells [157]. Therefore, the iron exporter FPN (ferroprotein) is reduced for breast cancer [158], prostate cancer [159], and ovarian [156], while its negative control, hepcidin, is adjusted upward [158,159]. Most cells including neoplastic cells store excess intracellular iron in ferritic cells, where it can be safely separated from participation in radical reactions [160]. Cancer cells increase metabolically available iron not only by increasing iron absorption and reducing its storage, but also by weakening its physiological function [161]. Studies have shown that overcharging with iron in the form of ferric ammonium citrate (FAC) or iron complexes significantly inhibits cell survival in different types of tumour [162]. Most patients with iron deficiency cancer have functional iron deficiency (FID), a condition with adequate iron stock, but insufficient iron supply for erythroblasts and other iron-dependent tissues [163]. Table 10 shows the iron concentrations tested in patients with oncological diseases (serum levels). Untreated excess iron can increase the risk of developing a disease, for example, liver cancer [164]. Serum ferritin (a protein that complexes Fe^3+^ and stores them in the liver) has been shown to be predictive in different cancers and elevated serum ft levels are associated with a poor prognosis [165,166,167]. The study by Sukiennicki et al. showed that serum iron levels could be a risk factor in larynx cancer and unselected patients with colorectal cancer [168]. The physiological level of iron is on average 70–170 µg/dL in the serum of healthy people [169]. Table 10 shows the level of iron concentration in patients with oncological diseases (serum levels).

Iron deficiency, caused by inflammatory processes associated with cancer or its treatment, and the use of ESA (erythropoiesis-stimulating agents), is quite common in cancer patients [171]. The incidence of anaemia in cancer patients is extremely high [163]. In patients with oral tumours, the addition of iron is ineffective because iron absorption by intestinal iron is significantly reduced and more than 95% of this element is excreted in faeces [163,172]. The treatment of iron deficiency in cancer is recommended by several specialised guidelines and should preferably be performed with intravenous iron administration [171]. Several studies have confirmed a significant correlation between the haemoglobin levels and the quality of life of cancer patients and physical efficiency [173,174]. Table 11 shows the reference values determined for copper.

Several approaches have been developed to treat cancer against intracellular iron metabolism disorders such as iron removal from cancer cells. Another way is to generate a cytotoxic level of ROS or ferroptosis by excess iron in neoplastic cells [165]. The European Society for Medical Oncology (ESMO) 2010 guidelines also recommend the periodic monitoring of iron homeostasis (iron, CRP, transferrin, and ferritin) [177]. The common guidelines of the American Society of Haematology/American Society of Clinical Oncology for the use of epoetin and darbepoetin in adult cancer patients (update 2010) require the monitoring of iron homeostasis at the beginning and during ESA treatment. If necessary, iron supplements should be used to improve the efficiency of ESA, reduce the necessary doses of ESA, and reduce the symptoms of the patient. However, there are insufficient data to recommend the optimal time and time intervals for the iron monitoring of Poles [178]. 

Oral preparations may consist of Fe(II) or Fe(III), except in Austria, where all available oral products contain only Fe(II), which is better tolerated [163]. In Europe, trace elements used in parenteral nutrition (PN) have been providing 1.0 to 1.2 mg per day for many years and contain Fe in the form of ferrous gluconate or ferric chloride [179]. Enteral nutrition should provide 18–30 mg of Fe per day with 1500 kcal [11]. An example of FSMP—Protein Nutrison^®^ Advance contains 2 mg of iron per 100 mL [62]. There is approximately 24 mg of Fe in a 1500 kcal Nutrison^®^ Protein Advance product. The product meets the 9.3 recompacts provided by ESPEN [11]. For patients receiving PN, the estimated need to maintain the iron level is 1 mg per day for adult men and women after menopause and approximately 2 mg per day for premenopausal women [180]. Iron deficiency is one of the most common complications of long-term PN—regular iron delivery is recommended using an iron supply [179]. A summary of the Fe content declared in FSMPs for oncological patients available on the EU markets is provided in Table 12.

### 3.5. Manganese

Manganese is a trace element necessary for proper human biology [181,182]. Mn is one of the most common metals in the human body, with a range of 0.3 to 2.9 mg of manganese per gram of wet tissue, mainly in the bones, liver, kidneys, pancreas, adrenal, and pituitary cells [183]. Manganese is an activator of numerous enzymes involved in the synthesis of proteins, nucleic acids, and fatty acids and is responsible for the regulation of blood sugar, cellular energy, and blood coagulation [184,185]. Its role in the regulation and transformation of thyroid hormones is indicated in [186]. The formation of antioxidant enzymes (e.g., MnSOD) is the body’s defence shield against radicals [187]. It is also necessary for the proper functioning of the nervous system, the brain, and pancreas, the formation of connective tissue and bone, and the normal condition of the skin [188]. High levels of manganese are usually found in the brain as a component of enzymes such as arginase, glutamine synthesis, phosphoenolipyromanic decarboxylase, pyrogenic carboxylases, and manganese superoxide dismutase enzymes [185]. The main route of manganese absorption is the gastrointestinal tract. Lung inhalation and intravenous infusion provide an additional avenue of absorption [11]. Manganese toxicity is more of a problem than deficiency [11]. Excess manganese exposure leads to the impairment of mitochondrial functions, oxidative stress, incorrect protein folding and transport, and inflammation of the nervous system [189]. The dietary intake of manganese does not cause toxicity because absorption is strictly regulated in the intestinal tract [183]. Patients suffering from cholestasis, liver failure, or liver spongiform encephalopathy can develop manganese toxicity because manganese is excreted in bile [190,191]. 

In the prevention and treatment of cancer, the influence of manganese compounds was investigated [192,193,194,195,196] in the work of Lv et al. Mn(II) was found to be essential in the detection of the innate immunity of cancerous tumours because Mn-deficient mice had significantly increased tumour growth and metastasis, with a greatly reduced number of tumour-infiltrating CD8 + T cells [194]. Mn is essential for anticancer immune responses. Mn-deficient mice were significantly more susceptible to B16F10 tumour invasion compared to the control mice, as indicated by a significant increase in the tumour size and weight [194]. Research on hollow mesoporous manganese oxides (HM–MON) has shown that they not only increase the therapeutic efficacy, but also perform multimodal cancer diagnosis [195]. Manganese toxicity varies depending on the route of exposure. When swallowed, manganese has relatively low toxicity at typical exposure levels and is considered as a trace element necessary for food [197]. Taking manganese as a nutritional factor into account, the EPA (Environmental Protection Agency) concluded that existing scientific information cannot determine whether excessive manganese can cause cancer [198]. The U.S. Environmental Protection Agency adopted a similar position and concluded that manganese is not classifiable as a human carcinogenic [184]. Significant changes in Mn were found in malignant colorectal cancer tissue compared to healthy tissue [199]. In [200], an inverse relationship was observed between the total Mn dietary intake and the risk of non-Hodgkin lymphoma. The reference range for manganese for healthy people is 0.7–0.12 µg/dL [201]. Table 13 summarises the data on Mn as a potential biomarker for different kinds of cancer.

Data in the literature on manganese levels in cancer patients are limited. For prostate cancer, the serum levels were significantly reduced compared to the controls [39]. IOM (2001) concluded that the concentration of manganese in serum/plasma or urine may be sensitive to high fluctuations in consumption. The concentration of manganese in whole blood appears to be very variable and has a limited value as an indicator of intake or status [15]. Manganese toxicity is often observed in adult patients, and the literature in the last 10 years has made various recommendations for the safe administration of manganese to these patients [204]. Toxicity was observed in adults receiving >500 mg/day and in paediatric patients receiving >40 mg/kg/day [205]. A dose of 110 mg/day in adults increases the concentration of manganese in the whole blood [206]. The estimated average consumption of manganese by adults in the EU ranges from 2 to 6 mg/day, most of which is about 3 mg/day. There may be large differences between individuals, depending on individual characteristics and eating habits (e.g., vegetarian diet vs. mixed diet) [187]. It is difficult to assess the manganese consumption or condition with biological markers due to the rapid withdrawal of manganese with bile, homeostatic control, and the lack of sensitivity of biomarkers in the normal range of consumption [187]. The EFSA Panel proposes an appropriate dietary intake (AI) for adults based on the average observed manganese intake observed from mixed diets in the EU. It was considered unnecessary to provide gender-related values [187]. Table 14 shows the values/indices designated for manganese.

To our knowledge, no manganese is added to food [209]. However, it is included in many nutritional supplements, some of which contain significant amounts of food, at a dose greater than 10 mg/day [210]. Research on Mn toxicity or its nutritional benefits is far from over and will become even more intensive in the next decade [197]. Dietary intake does not cause toxicity because absorption is strictly regulated in the intestine [211]. The UL for manganese in the diet is 11 mg/d for adults [15]. Manganese toxicity is related to environmental and occupational exposure [211]. Manganese toxicity may also be due to certain conditions and PN [5,197,212]. According to ESPEN, the micronutrient guideline is provided in PN preparations as both an essential element in the range of 1 to 10 mmol (55–550 mg)/day and as an impurity. It is now clear that intravenous intake of 2 mmol (110 mg)/day is excessive during PN. Therefore, there is currently evidence of 1 mmol (55 mg) per day for patients who receive PN. Manganese contamination should be limited to less than 40 mg/d total in a typical adult PN formulation [11]. The increase in Mn consumption was negatively related to the risk of liver cancer and the low consumption of Mn-rich foods such as leafy vegetables, whole cereals, nuts, legumes, and tea for liver cancer [213]. Intake should be 2–3 mg of manganese per day, but doses of up to 6 mg/day administered at 1500 kcal of FSMP are considered safe [11]. A summary of the Zn content declared in the FSMPs for oncological patients available on the EU markets is provided in Table 15.

As can be seen, the manganese content declared by the producers in the products for patients with special medical oncology patients is different. Individual FSMPs contain approximately 0.25 mg/100 mL–1.3 mg/100 mL Mn, while some of them do not have a specific critical element content.

## 4. Conclusions

Based on a critical review of the elements investigated and analysed in the FSMPs for oncology patients available on the EU markets, appropriate conclusions can be drawn.

- Disturbances in Zn homeostasis are observed in oncology patients, therefore, the appropriate level of this essential element should be controlled during nutritional treatment with FSMPs, according to the ESPEN micronutrient guidelines;
- Based on a review of the literature, there is evidence that the Cu level is correlated with cancer stage, therefore, the supply and bioavailability of copper from FSMPs should be verified to avoid falsifying test results for cancer recurrence;
- In light of the data and guidelines of the current literature, the use of different amounts of Se in FSMPs seems to be safe;
- Fe, due to its important function in the body and significant changes in metabolism during cancer, deserves special attention in the correlation of supply with FSMPs and the state of iron metabolism (e.g., serum level);
- Mn is not recognised as a carcinogenic factor, it participates in the immune response; therefore, it can be used safely in oncological patients fed with FSMPs. Additionally, a deficiency in this element in the diet of cancer patients should be monitored.

Regarding the summary of elements in the analysis of the FSMPs, the following conclusions can be drawn.

- There are no clear and unambiguous technical guidelines for producers (qualitative and quantitative) on the element (Zn, Cu, Se, Fe, Mn) content of the FSMPs for oncology patients;
- Manufacturers do not verify or comply with the scientific literature on the requirements of FSMPs for cancer treatment;
- FSMP manufacturers do not verify that elements that are in excess or under measurement may be additional carcinogens in oncology patients;
- FSMPs for cancer patients should have separate technical and quality guidelines and be seen as a pharmaceutical product;
- Manufacturers use different ways of marking FSMPs (e.g., different units of element content), therefore, the legal implications of the introduction of the use of the same unit should be considered;
- Manufacturers of FSMPs do not consider that a product without an element in its composition may be contaminated (e.g., during many processes);
- When determining the composition of FSMPs for oncological patients, the supply of the element in the full-day food ration should be considered.

The importance of element concentration and content in feeding oncology patients with FSMPs is not overestimated. Some of the FSMP products do not have a specific content of individual elements. In general, the legal regulations and recommendations on the content of elements in FSMP require a systematic and up-to-date approach including holistic and toxicological approaches to patients and the composition of PN nutrition products. Cooperation with toxicology, dietetics, medical doctors, manufacturers, and other medical personnel should be undertaken to establish uniform and unambiguous recommendations on foods for particular nutritional uses. Labelling foods for particular nutritional uses should be a simple and intuitive way for patients and medical staff. Across the community of cancer specialists, audit changes in the FSMP manufacturing practice are needed to ensure the safe and complete feeding of cancer patients through FSMPs.

## Figures and Tables

**Table 1:** Zn as a potential biomarker of different kinds of cancer—critical review.

Type of Cancer	Type of Change, Biological Material, Value [µg/dL]	Reference(s)
Cancer cachexia	Normal, serum, 71.00	[32]
Breast cancer	Normal, serum, 110.96	[33]
Gastric cancer	Increase, serum, 233.00	[34]
Endometrial cancer	Increase, serum, 183.00	[35]
Lung cancer	Increase, serum, 85.00–183.00	[36]
Pulmonary cancer	Increase, serum, 248.00	[37]
Gastrointestinal cancer	Increase, serum, 273.00	[37]
Gynaecological cancer	Increase, serum, 249.00	[37]
Colorectal Cancer CRC	Normal, serum, 78.00–97.00	[38]
Prostate cancer	Decrease, serum, 51.00	[39]

**Table 2:** Important dietary indices/parameters for Zn based on literature review.

Parameter/Index	Value	Reference(s)
RDA/PRI (mg/day)	8–11	[44]
EAR/AR (mg/day)	6.5–12	[43,44]
UL (mg/day)	40	[44,45]
RDI (mg/day)	8.0–14.0	[44]
ADI (mg/day)	14–20	[46]
PMTDI (mg/kg bw/d.)	0.3–1	[46,47]
EDI (μg/day)	10,496–13,459	[47]
DRV (mg/day)	7.5–16.3	[43,48]

**Table 3:** Summary of the Zn content declared in the FSMP for oncological patients available on the EU markets.

Declared Zn Content	Product	Source
1.3 mg/100 mL	Resource^®^ Protein	[61]
1.2 mg/100 mL	Nutrison^®^	[62]
1.2 mg/100 mL	Nutrison^®^ Multi Fibre	[63]
1.2 mg/100 mL	Nutrison^®^ Soya	[64]
1.8 mg/100 mL	Nutrison^®^ 1000 Complete Multi Fibre	[64]
1.5 mg/100 mL	Nutrison^®^ Protein Plus	[65]
1.7 mg/100 mL	Resource^®^ 2.0	[66,67]
1.7 mg/100 mL	Resource^®^ 2.0 + Fibre	[68]
ND	Resource^®^ Refresh	[69]
1.5 mg/100 mL	Resource^®^ Diabet Plus	[70]
ND	Resource^®^ Instant Protein	[71]
0.9 mg/100 mL	Nutramil^®^ Complex	[72]
2.0 mg/200 mL	Nutramil complex^®^ Protein	[73]
1.2 mg/100 mL	Fortimel Pulver	[74]
1.9 mg/100 mL	Provide Xtra Drink	[75]
1.8 mg/100 mL	Survimed^®^ OPD	[76]

**Table 4:** Copper as a potential biomarker of different types of cancer based on a critical review.

Type of Cancer	Type of Change, Biological Material, Value [µg/dL]	Reference(s)
Breast cancer	Increase, serum, 137.15 ± 36.24 Increase, serum, 128.15 ±19.14	[33] [101]
Endometrial cancer	Increase, serum, 372 ± 215	[35]
Thyroid cancer	Normal, serum, 6.2 ± 0.9	[102]
Lung cancer	Increase, serum, 127 ± 27	[103]
Cervical cancer	Increase, serum, 147 ± 26 Increase, serum, 156.9 ± 3.4	[104] [105]
Colorectal Cancer CRC	Increase, serum, 123.75 ± 27.11	[38]
Oral cancer	Increase, serum, 95.85	[106]
Lung cancer	Increase, serum, 176.00	[36]
Prostate cancer	Increase, serum, 169.00	[39]

**Table 5:** Important dietary indices/parameters for copper as an essential element.

Parameter/index	Value	Reference(s)
RDA/PRI (mcg/day)	200–900	[108]
EAR/AR (mg/day)	ND *	[96]
AI (mg/day)	1.3–1.6	[96]
UL (mg/day)	1.0–10.0	[109]
RDI/DRI (mg/day)	ND	ND
ADI (mg/kg bw/d.)	0.07	[96]
PMTDI (mg/kg bw/d.)	ND	ND

* Due to the lack of suitable copper biomarkers and the limitations of available equilibrium studies, EFSA was unable to determine the average demand (AR) [96].

**Table 6:** Summary of the Cu content declared in FSMPs for oncological patients available on the EU markets.

Declared Cu Content	Product	Source
0.29 mg/100 mL	Nutricia^®^ FortiCare	[120]
0.18 mg/100 mL	Nutricia^®^ Nutrison	[77]
0.21 mg/100 mL	Nutricia^®^ Nutrison Diason Energy HP	[78]
0.27 mg/100 mL	Nutricia^®^ Nutrison 1000 Complete Multi Fibre	[121]
0.15 mg/100 mL	Resource^®^ Protein	[122]
ND	Resource^®^ Refresh	[69,123]
0.13 mg/100 mL	Resource^®^ Diabet Plus	[70,124]
0.75 mg/100 mL	Fortimel Pulver	[74,125]
0.09 mg/100 mL	Nutramil^®^ Complex	[72]
0.20 mg/200 mL	Nutramil complex^®^ Protein	[73]
0.38 mg/100 mL	Provide Xtra Drink	[75]
0.20 mg/100 mL	Survimed^®^ OPD	[126]

**Table 7:** Se as a potential biomarker of different types of cancer based on a critical review.

Type of Cancer	Type of Change, Biological Material, Value [µg/dL]	Reference(s)
Laryngeal cancer	Decrease, serum, 5.87	[141]
Prostate cancer	Normal, serum, 12.56	[142]
Breast cancer	Decrease, serum, 8.62	[143]
Lung cancer	Decrease, serum, 7.86	[144]
Prostate cancer	Decrease, serum, 7.00	[39]

**Table 8:** Important dietary indices/parameters for Se based on a critical review.

Parameter/Index	Value	Reference(s)
RDA/PRI (μg/day)	55	[147]
EAR/AR (μg/day)	45	[147]
UL (μg/day)	400	[148]
RDI/DRI	70	[149]
ADI (μg/day)	ND	ND
PMTDI (mg/kg bw/d.)	ND	ND
EDI (μg/day)	ND	ND

**Table 9:** Summary of the Se content declared in FSMPs for oncological patients based on the products available on the EU markets.

Declared Se Content	Product	Source
10 μg/100 mL	Survimed^®^ OPD	[126]
4.8 μg/100 mL	Nutramil^®^ Complex	[72]
12 μg/100 mL	Resource^®^ 2.0 + Fibre	[68]
7.5 μg/100 mL	Resource^®^ Protein	[71]
5.7 μg/100 mL	Nutrison^®^ Soya	[150]
8.5 μg/100 mL	Nutrison^®^ Multi Fibre	[121]
5.7 μg/100 mL	Nutrison^®^	[77]
8.5 μg/100 mL	Nutrison^®^ 1000 Complete Multi Fibre	[121]
3.5 μg/100 mL	Fortimel Pulver	[125]
12.5 μg/100 mL	Provide Xtra Drink	[75]
7.1 μg/100 mL	Nutrison^®^ Protein Plus	[65]
12 μg/100 mL	Resource^®^ 2.0	[66]
10 μg/100 mL	Resource^®^ Diabet Plus	[124]
11 μg/100 mL	Nutramil Complex^®^ Protein	[73]

The FSMPs in Table 9 contain 2.6–12.5 µg/100 mL [64,65,69,70,71,73,118,121,122,123,146].

**Table 10:** Iron as a potential biomarker of different types of cancer based on a critical review.

Type of Cancer	Type of Change, Serum Levels, Value [µg/dL]	Reference(s)
Liver cancer	Normal, serum, 142	[170]
Lung cancer	Normal, serum, 150	[170]
Kidney cancer	Normal, serum, 169	[170]
Breast cancer	Normal, serum, 81	[170]
Colorectal cancer	Normal, serum, 12	[170]
Prostate cancer	Normal, serum, 196	[39]

**Table 11:** Important dietary indices/parameters for Fe reported in the present review.

Parameter/Index	Value	Reference(s)
RDA/PRI (mg/day)	11	[152]
EAR/AR (mg/day)	6	[175]
UL (mg/day)	45	[176]
RDI/DRI (mg/day)	8	[11]
ADI (μg/day)	ND	ND *
PMTDI (mg/kg bw/day)	ND	ND *
EDI (μg/day)	ND	ND *

* no data.

**Table 12:** Summary of the Fe content declared in the FSMPs for oncological patients available on the EU markets.

Declared Se Content	Product	Source
1.3 mg/100 mL	Survimed^®^ OPD	[126]
0.65 mg/100 mL	Nutramil^®^ Complex	[72]
1.5 mg/100 mL	Resource^®^ 2.0 + Fibre	[68]
1.5 mg/100 mL	Resource^®^ Protein	[71]
1.6 mg/100 mL	Nutrison^®^ Soya	[150]
2.4 mg/100 mL	Nutrison^®^ Multi Fibre	[121]
1.6 mg/100 mL	Nutrison^®^	[77]
2.4 mg/100 mL	Nutrison^®^ 1000 Complete Multi Fibre	[121]
3.5 mg/100 mL	Fortimel Pulver	[125]
2.5 mg/100 mL	Provide Xtra Drink	[75]
2 mg/100 mL	Nutrison^®^ Protein Plus	[65]
2.4 mg/100 mL	Resource^®^ 2.0	[66]
1.2 mg/100 mL	Resource^®^ Diabet Plus	[124]
1.5 mg/100 mL	Nutramil Complex^®^ Protein	[73]

**Table 13:** Mn as a potential biomarker of a different kinds of cancer based on a critical review.

Type of Cancer	Type of Change, Biological Material, Value [µg/dL]	Reference(s)
Prostate cancer	Normal, serum, 0.100	[39]
Breast cancer	Increase, serum, 0.175	[202]
Colorectal cancer	Increase, serum, 0.770	[203]

**Table 14:** Important dietary indices/parameters for Mn reported in the present review.

Parameter/Index	Value	Reference
AI (mg/day)	3	[187]
RDA/PRI (mg/day)	ND *	[187]
EAR/AR (mg/day)	ND *	[187]
UL (mg/day)	11	[207]
RDI/DRI (mg/day)	ND	ND
ADI (μg/day)	ND	ND
PMTDI (mg/kg bw/day)	0.5	[208]
EDI (μg/day)	ND	ND
PTWI (mg/kg bw/week)	3.5	[208]

* The EFSA Panel concludes that there is insufficient evidence to determine the average consumption (AR) and the consumption of manganese in the reference population (PRI) [187].

**Table 15:** Summary of the Mn content declared in the FSMP for oncological patients available on the EU markets.

Declared Mn Content	Product	Source
0.28 mg/100 mL	Resource^®^ Protein	[122]
0.33 mg/100 mL	Nutrison^®^	[77]
0.50 mg/100 mL	Nutrison^®^ Multi Fibre	[121]
0.33 mg/100 mL	Nutrison^®^ Soya	[150]
0.50 mg/100 mL	Nutrison^®^ 1000 Complete Multi Fibre	[121]
0.41 mg/100 mL	Nutrison^®^ Protein Plus	[65]
0.25 mg/100 mL	Resource^®^ 2.0	[206]
0.32 mg/100 mL	Resource^®^ 2.0 + Fibre	[68]
ND	Resource^®^ Refresh	[123]
0.26 mg/100 mL	Resource^®^ Diabet Plus	[124]
ND	Resource^®^ Instant Protein	[71]
0.17 mg/100 mL	Nutramil^®^ Complex	[72]
0.2 mg/100 mL	Nutramil complex^®^ Protein	[73]
0.65 mg/100 mL	Fortimel Pulver	[74]
0.5 mg/100 mL	Provide Xtra Drink	[75]
0.27 mg/100 mL	Survimed^®^ OPD	[126]

## Data Availability

The data, analytic methods, and study materials that support the findings of this study are available from Kamil Jurowski (kjurowski@ur.edu.pl) upon reasonable request.

## Abbreviations

ADI—acceptable daily intake; AI—adequate intake; AR—average requirement; CRC—colorectal cancer; CRP—C-reactive protein; DRV—dietary reference values; DRV—dietary reference values; EAR—estimated average requirement; EDI—estimated daily intake; EFSA—European Food Safety Authority; ESA—erythropoiesis stimulating agents; ESPEN—European Society for Clinical Nutrition and Metabolism; FAC—ferric ammonium citrate; FID—functional iron deficiency; FPN—ferroprotein; FPN—ferroprotein; FSMP—food for special medical purposes; IFL—chemotherapy regimen for certain cancers, consisting of simultaneous treatment of irinotecan, leucorine (folic acid) and fluorouracil; PMTDI—provisional maximum tolerable daily intake; PN—parenteral nutrition; PRI—primary rate interface; PSA—prostate-specific antigen; RDA—recommended dietary allowances; RDI—reference daily intake; TM—tetrathiomolybdate; UL—tolerable upper intake level; WD—Wilson’s disease.
